# Supplementary material for: Yiai Fuzheng decoction inhibits triple-negative breast cancer by remodeling the immune microenvironment
Source: Front Immunol. 2025 Sep 30;16:1615631. doi: 10.3389/fimmu.2025.1615631 (PMC12518410; doi:10.3389/fimmu.2025.1615631)
Supplement: Supplementary file 2 [file DataSheet1.zip › Supplementary File 2/Treatment/C_vs_D_vs_H/06_Pathway_Analysis/Pathway_Analysis/Sig_Pathways_Name/Selenoamino acid metabolism.pdf]

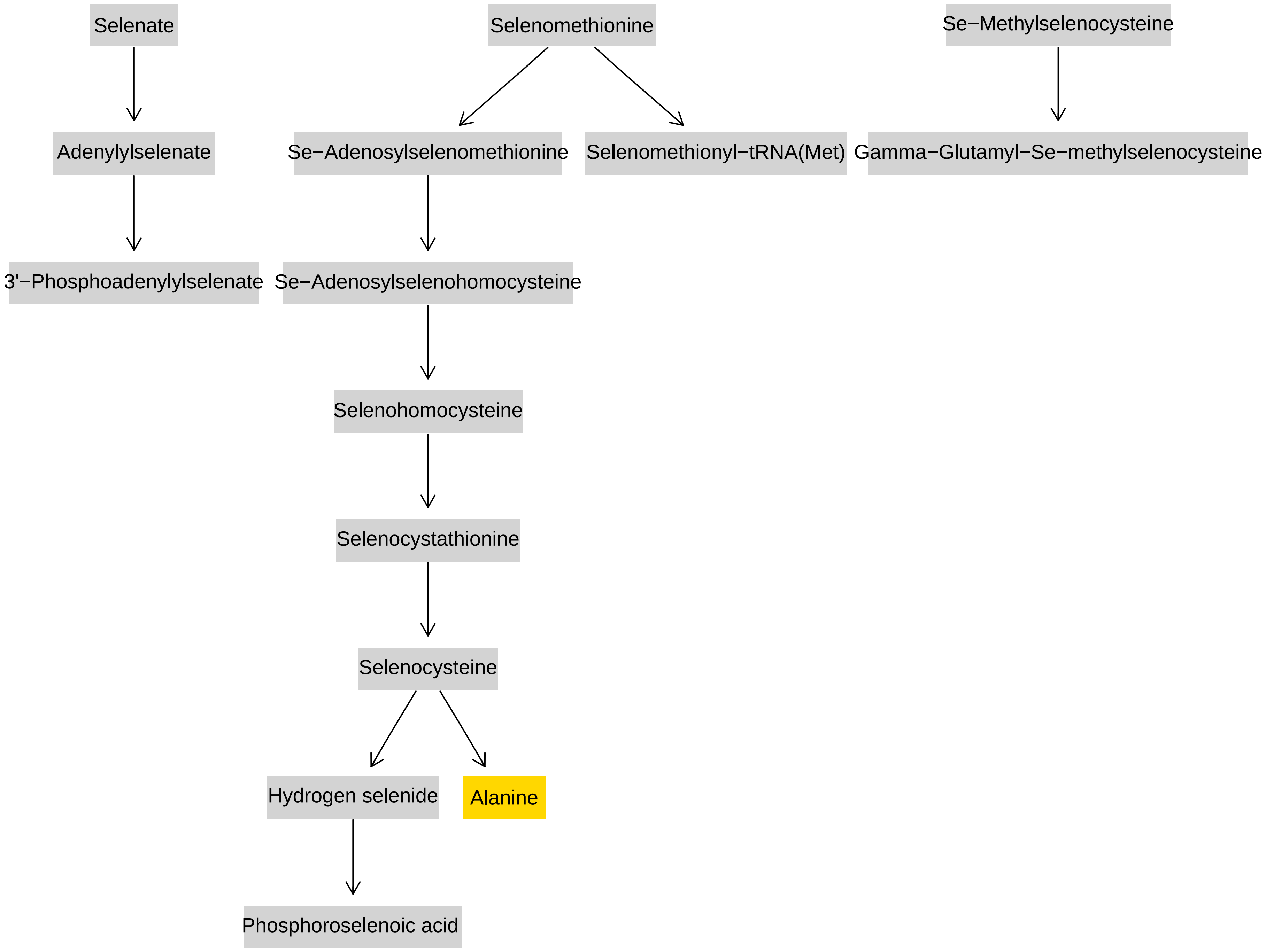
Selenate
Adenylylselenate
3'−Phosphoadenylylselenate
Selenomethionine
Se−Adenosylselenomethionine
Selenomethionyl−tRNA(Met)
Se−Adenosylselenohomocysteine
Selenohomocysteine
Selenocystathionine
Selenocysteine
Hydrogen selenide
Alanine
Phosphoroselenoic acid
Se−Methylselenocysteine
Gamma−Glutamyl−Se−methylselenocysteine
